# Regional Thermal Radiation Characteristics of FY Satellite Remote Sensing Based on Big Data Analysis

**DOI:** 10.3390/s23208446

**Published:** 2023-10-13

**Authors:** Tao Wen, Congxin Wei, Zhenyi Wang, Linzhu Wang, Zihan Yang, Tingting Gu

**Affiliations:** 1 Gansu Computing Center, Lanzhou 730030, China; went@cc.gs.cn (T.W.); wanglz@cc.gs.cn (L.W.);; 2 School of Computer and Communication, Lanzhou University of Technology, Lanzhou 730050, China; 3 Gansu Zijin Cloud Big Data Development Co., Ltd., Jinchang 737100, China

**Keywords:** satellite thermal radiation remote sensing, multiscale time-frequency relative power spectrum analysis, thermal radiation background, thermal anomaly

## Abstract

It is of great significance to study the thermal radiation anomalies of earthquake swarms in the same area in terms of selecting abnormal characteristic determination parameters, optimizing and determining the processing model, and understanding the abnormal machine. In this paper, we investigated short-term and long-term thermal radiation anomalies induced by earthquake swarms in Iran and Pakistan between 2007 and 2016. The anomalies were extracted from infrared remote sensing black body temperature data from the China Geostationary Meteorological Satellites (FY-2C/2E/2F/2G) using the multiscale time-frequency relative power spectrum (MS T-FRPS) method. By analyzing and summarizing the thermal radiation anomalies of series earthquake groups with consistency law through a stable and reliable MS T-FRPS method, we first obtained the relationship between anomalies and ShakeMaps from USGS and proposed the anomaly regional indicator (ARI) to determine seismic anomalies and the magnitude decision factor (MDF) to determine seismic magnitude. In addition, we explored the following discussions: earthquake impact on regional thermal radiation background and the relationship between thermal anomalies and earthquake magnitude and the like. Future research directions using the MS T-FRPS method to characterize regional thermal radiation anomalies induced by strong earthquakes could help improve the accuracy of earthquake magnitude determination.

## 1. Introduction

The application of satellite remote sensing thermal radiation data to study earthquake anomalies and mechanisms is one of the research directions of geophysics, which has obtained many research results. One of the earliest studies on the relationship between satellite radiation remote sensing and earthquakes dates back to the 1980s [1,2]. With the development of satellite remote sensing technology, many efforts have been devoted to studying the earthquake thermal radiation characteristics. It has been reported that significant thermal anomalies can be identified prior to earthquakes in seismically active areas [2,3,4,5,6,7,8,9,10,11]. Such examples include the following: Bhuj (Gujarat), Mw = 7.7, 26 January 2001, India [6,11]; Colima, Mw = 7.8, 21 January 2003, Mexico [11]; Kashmir, Mw = 7.6, 8 October 2005, [3]; Bhuj (Gujarat), Mw = 7.7, 26 January 2001, India [6,10]; Colima, Mw = 7.8, 21 January 2003, Mexico [11]; Yutian, Mw = 7.3, 21 March 2008, China [12]; Wenchuan, Mw = 8.0, 12 May 2008, China [13]; the Andaman Islands, Mw = 7.5, 11 August 2009 [14]; Honshu, Mw = 9.1, 11 March 2011, Japan [15]; and many others.

However, in seismic thermal radiation research, anomaly extraction is difficult because of the weak signals and complicated background noise. First of all, satellite remote sensing images were used in structural geological and geomorphologic research. Active faults and structures were mapped on the base of satellite images. This method is very limited in time series analysis. There was no possibility to measure short-term processes before and after the earthquake. It was as simple as an extrapolation of air photo geological interpretation methods applied to space. The modern version of this method is active tectonic analysis with the application of alignment analysis. Case studies of various thermal remote sensing application methods for earthquakes have been recently reported. The use of MISR (Multi-angle Imaging Spectra Radiometer) data revealed a significant increase in surface moisture after the Bhuj (Gujarat) earthquake on 26 January 2001. This indicates that changes in surface moisture after an earthquake can be detected through thermal remote sensing technology [16]. The discovery that anomalous areas are consistent with seismic zones or active tectonics, with epicenters often located at the intersections of stress tropics, suggests a certain correlation between seismic activity and thermal remote sensing anomalies, possibly related to tectonic activity [17,18,19,20,21]. The analysis of satellite thermal infrared brightness temperature anomaly images reveals that short-term anomalies exhibit clearly identifiable radiation characteristics. This suggests that by analyzing radiation anomalies at short-term time scales, specific feature information can be obtained [22]. The study of the temporal-spatial variations of associated faulting inferred from satellite infrared information reveals a close correlation between the Mani earthquake radiation anomaly and the activity of the Altyn Tagh fault, illustrating the interactions between faults and defection among the deformation exception, current fault activities, and earthquakes [23]. Secondly, time series of alignment distributions on the Earth’s surface are investigated before and after an earthquake. Tramutoli and colleagues have developed a robust satellite data analysis technique for space–time thermal anomalies on the Earth’s surface recorded by satellites months to weeks before the occurrence of earthquakes. Lastly, since 2000, Zhang et al. have researched anomaly extraction using case studies and have investigated underlying mechanisms [24]. They developed the time-frequency relative power spectrum (T-FRPS) method based on radiation brightness temperature remote sensing data from the China Geostationary Meteorological Satellites (FY-2C/2E/2F/2G) and proposed the ‘union origin mechanism’ to explain pre-earthquake thermal anomalies [25]. The thermal radiation anomaly characteristics, including characteristic period, amplitude, duration, and morphology, were clear and valuable for pre-earthquake thermal radiation research. The epicenters were located near the intersection of the anomaly migration processes, which was consistent with the remote sensing rock test mechanics [26,27].

Researchers have achieved certain results in the extraction methods of seismic anomaly information (from viewing pictures to time series and time-frequency analysis) and earthquake case studies. However, there are few studies in some directions, such as the characteristics of thermal radiation anomalies and the characteristics of swarms of earthquakes over many years; there are many studies on short-term and anomalous thermal radiation, but there are few studies on mid- and long-term background anomalies, and thermal radiation background characteristics, as well as the characteristics of thermal radiation backgrounds and the impacts of strong earthquakes on thermal radiation background field.

Therefore, this article details the multiscale time-frequency relative power spectrum (MS T-FRPS), it initially scrutinizes the results of Wavelet filtering results, and conducts an annual background anomaly analysis of Mw 7+ earthquakes. Additionally, it extracts short- and long-term thermal radiation anomalies of several strong earthquake swarms in Iran and Pakistan. Furthermore, it compares and analyzes the similarities and differences between short-term and long-term background abnormalities for the first time. The impacts of strong earthquakes on thermal radiation background field are also discussed for the first time.

## 2. Methodology

### 2.1. Multiscale Time-Frequency Relative Power Spectrum (MS T-FRPS) Analysis

Time-frequency analysis (TFA) is a powerful tool for analyzing time-varying non-stationary signals and has received increasing attention in recent years. TFA provides joint distribution information of the time domain and the frequency domain, which clearly describes the signal frequency over time. The basic idea of TFA is to design a joint function of time and frequency that simultaneously describes the energy density or intensity of the signal at different times and frequencies. This joint function of time and frequency is referred to simply as the time-frequency distribution, by which we can draw the instantaneous frequency and amplitude at each moment and conduct time-frequency filtering and time-varying analysis of the signal.

Firstly, Daubechies wavelet transform (2nd-order scale and 7th-order scale filtering) is used to process the multi-year data of satellite thermal radiation daily values to separate the temperature changes caused by the basic temperature field, the annual temperature field, and other factors. In addition, the 6th- and 7th- order scales are respectively decomposed to obtain the annual and approximate annual background information. Secondly, the Welch’s FFT power spectrum is applied to process the above wavelet filtering and decomposition results, in order to obtain the dominant frequency and amplitude in the time domain. Therefore, the method of successive wavelet processing and power spectrum processing is called time-frequency power spectrum analysis. Thirdly, in order to compare and analyze the power spectrum of the thermal radiation change before and after the earthquake, the power spectrum values of all the frequencies of each pixel are relatively processed to generate spatial-temporal data of the relative time-frequency power spectrum. The frequency analysis method through wavelet transform at different scales (6th-order scale, 7th-order scale, and 2nd- and 7th-order bandpass filtering) and relative Fourier power spectrum calculation for different window lengths (64 days, 128 days, 256 days, and 512 days) is called multiscale time-frequency relative power spectrum (MS T-FRPS).

#### 2.1.1. Wavelet and Fourier Transform

Wavelet theory is a relatively new digital signal processing technology that originated a few decades ago. Due to its high resolution in both the time and frequency domains, it is called the ‘mathematical microscope’, which has been widely used and is developing rapidly in the field of digital signal processing. All wavelet transforms may be considered as forms of time-frequency representation for continuous-time signals and thus are related to harmonic analysis. Almost all practically useful discrete wavelet transforms use discrete-time filter banks, which are called wavelet and scaling coefficients in the wavelet nomenclature. The most commonly used set of discrete wavelet transforms was formulated by Ingrid Daubechies in 1988 [28]. The Daubechies wavelets are a family of orthogonal wavelets, with D2-D20 and db1-db10 commonly used. In this paper, the db8 wavelet basis function of the Daubechies (dbN) wavelet system is used as the basic transform window function to process daily value brightness temperature data.

Spectral analysis is an important method for studying signal characteristics. For deterministic signals, Fourier transform (FT) can be used to examine its spectral properties. Any signal that can be represented by amplitude that varies in time has a corresponding frequency spectrum, which contains all the information of the original signal. This means that the original function can be completely reconstructed by an inverse FT. In classical spectral estimation using a periodical diagram, the variance of spectral estimate at a specific frequency does not decrease with the increasing number of samples. Although it is successful in examining the frequency characteristics of noise-free functions, it cannot process noise-like signals, e.g., sinusoids with low signal-to-noise ratios. To mitigate this issue, Welch modified the periodic diagram method by taking average over time, which becomes popular for estimating the power of a signal at different frequencies. Due to its ease of understanding and calculation, it has been widely used and adopted in our analysis.

#### 2.1.2. Relative Processing

In order to obtain the relative time-frequency power spectrum of each pixel, we calculate it by Equation (1),

(1) $$R_{PS}=\frac{R_{i,j}}{R_{i,Mean}}$$

where $R_{PS}$ is the calculated relative power spectrum value, $R_{i,j}$ is the power spectrum value of a certain pixel at any time, and $R_{i,Mean}$ is the ten-year average of the corresponding pixel.

### 2.2. Data Acquisition

Geostationary meteorological satellites are preferable to polar orbit satellites for earthquake research as they consistently observe the same location. We used radiation brightness temperature remote sensing data acquired from the China Geostationary Meteorological Satellites FY-2C/2E/2F/2G, which were launched during 2004–2014. The scan period was 30 min or 1 h, and the spatial resolution was 5 km. The data were collected from 1 January 2007 to 31 December 2016. Five midnight observations were selected from each day because of the lower solar radiation at night.

### 2.3. Data Processing

In this paper, the wavelet transform basis function and the relative FT are designed as a joint function of time and frequency, which describes the energy density or intensity of the signal at different times and frequencies. The TFA function was used to analyze the similarities and differences between time-frequency analyses at each scale.

**Figure 1:**
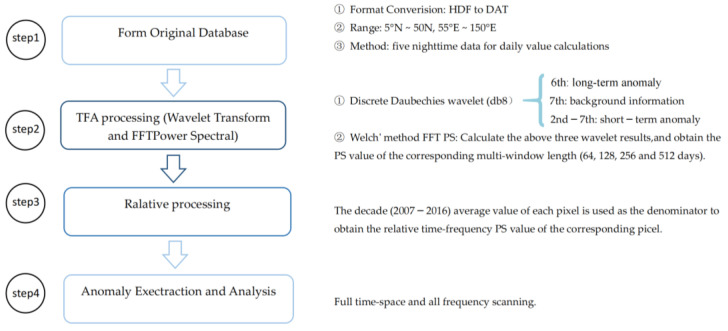
Data processing flowchart.

Data processing and extraction were conducted in four steps, as shown in Figure 1:

1. The original database was formed after pre-processing the radiation remote sensing black body temperature data from the Chinese geostationary meteorological satellite. This process included data format conversion, data space range interception, removal of clouds, and selection of nighttime data for daily value calculations. The influence of partial cloud cover was removed by making up the window. Then, we calculated the mean value of five data points to obtain the daily value.
2. Various scale-factor transformation results were obtained by applying discrete Daubechies wavelet transformations. And the dominant frequency and the peak-to-peak amplitude values were obtained using the FFT power spectral method.
3. The decade (2007–2016) average value of each pixel was used as the denominator to obtain the relative time-frequency PS value of the corresponding pixel.
4. Time-frequency mapping was used to extract earthquake thermal radiation anomaly data. The non-seismic factors were effectively removed and earthquake thermal radiation anomalies were highlighted by scanning the time-frequency space data over the full spatial-temporal and band range.

## 3. Study Area

### 3.1. Regional Tectonic Background

**Figure 2:**
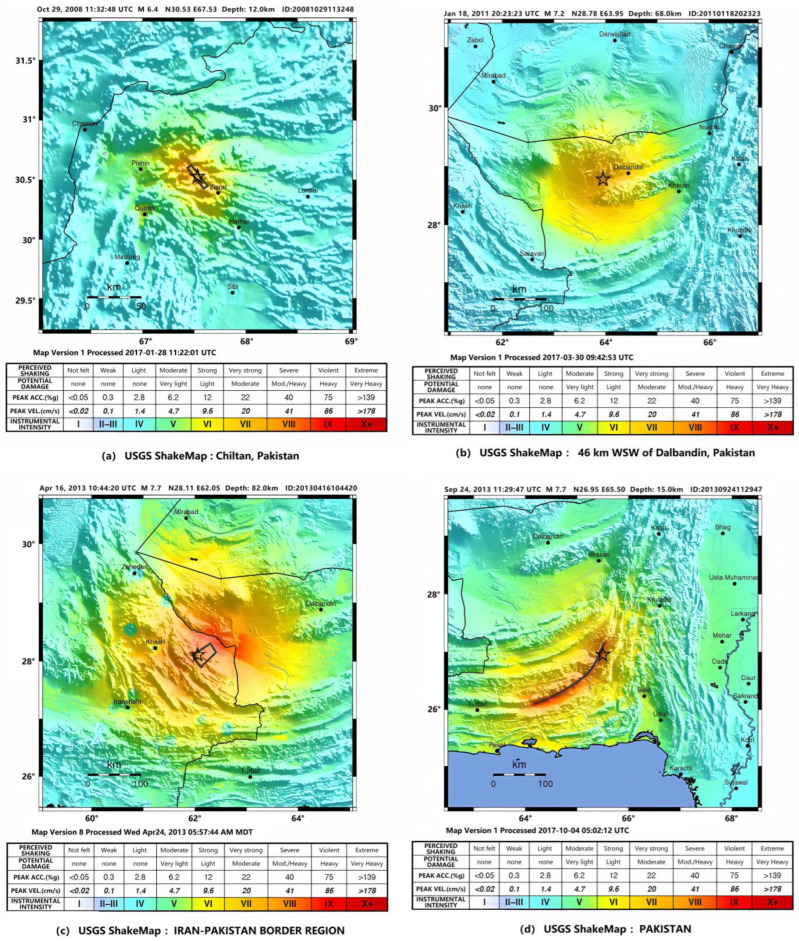
ShakeMap of four earthquakes. Source: United States Geological Survey.

The tectonics of this investigated region is dominated by the collisions of the Arabian and Indian plates with Eurasia. The Arabian plate lithosphere subducts beneath the Eurasian plate at the Makran coast of Pakistan and Iran, and extends progressively deeper to the north [29]. Four major tectonic plates (Arabia, Eurasia, India, and Africa) and one smaller tectonic block (Anatolia) are responsible for seismicity in the Middle East and surrounding region. The geologic evolution of this region is a consequence of several first-order plates’ tectonic processes including seductions, large-scale transform faulting, compression mountain building, and crustal extension. Continental thickening of the northern and western edge of the India subcontinent has produced the highest mountains in the world, including the Himalayan, Karakoram, Pamir, and Hindu Kush ranges. Earthquake activity and faulting found in this region as well as adjacent parts of Afghanistan and India are due to collisional plate tectonics. Shallower crustal earthquakes in the Pamir-Hindu Mountains occur primarily along the Main Pamir Thrust and other active Quaternary faults, which accommodate much of the region’s crustal shortening. From 2007 to 2016, 4 earthquakes with Mw 6.0+ occurred in this region; the ShakeMaps of the main shocks are shown in Figure 2 and the source is the following website: (a) https://earthquake.usgs.gov/earthquakes/eventpage/usp000gma2#shakemap, (accessed on 1 July 2023); (b) https://earthquake.usgs.gov/earthquakes/eventpage/usp000htb4#shakemap, (accessed on 1 July 2023); (c) https://earthquake.usgs.gov/earthquakes/eventpage/usb000g7x7#shakemap, (accessed on 1 July 2023); (d) https://earthquake.usgs.gov/earthquakes/eventpage/usb000jyiv#shakemap, (accessed on 1 July 2023). In Figure 2, the star marks the epicenter, and the lines around the star represent the rupture direction of the earthquake.

### 3.2. Regional Earthquake Activity History

**Figure 3:**
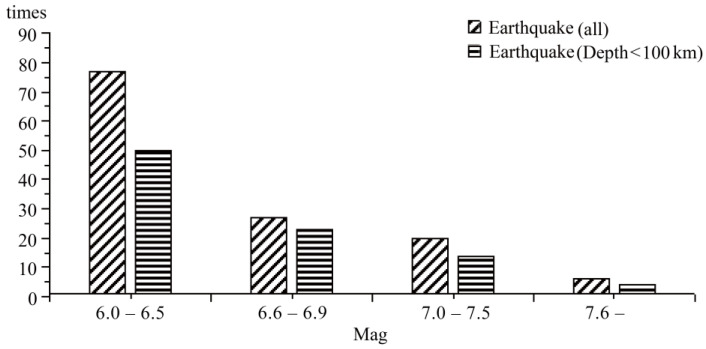
Regional MAG time map from 1900 to 2016. Region: 22–38° N, 55–71° E, earthquake magnitudes: Mw 6+; source: United States Geological Survey.

This region had strong seismic activity from 1900 to 2016, including 130 earthquakes (100 shallow earthquakes) of magnitude 6+, as shown in Figure 3, and the source is the following website: https://earthquake.usgs.gov/earthquakes/search/, accessed on 10 July 2023, Date: 1 January 1990–31 December 2016. The number of earthquakes decreased as magnitude increased. Previous studies have found that the thermal radiation anomalies of shallow earthquakes are more obvious and easier to extract. Therefore, we focused on analyzing shallow-source seismic activity.

**Figure 4:**
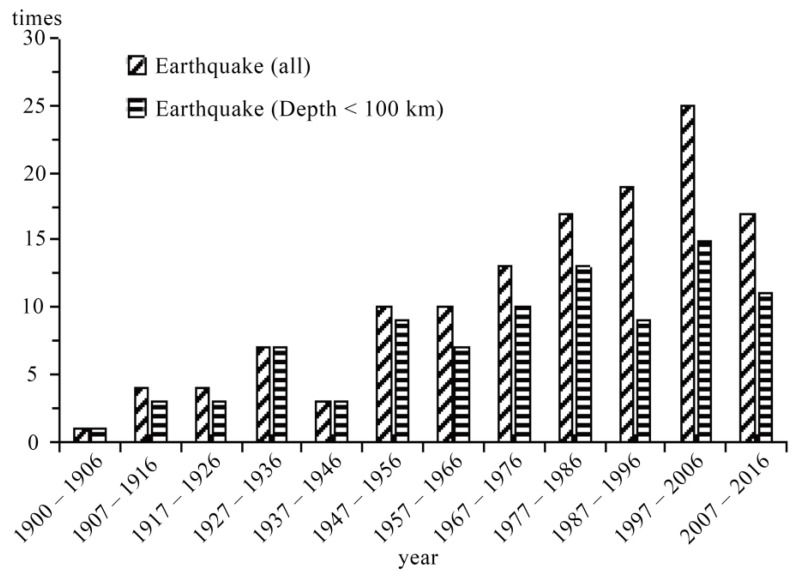
Regional decade interval earthquake frequency chart from 1900 to 2016. Region: 22–38° N, 55–71° E; earthquake magnitude: Mw 6+; source: United States Geological Survey.

Since 1900, the frequency of earthquakes per decade has gradually risen, peaking between 1997 and 2006, as shown in Figure 4, and the source is the following website: https://earthquake.usgs.gov/earthquakes/search/, accessed on 10 July 2023, Date: 1 January 1990–31 December 2016. Earthquakes occurred less frequently before 1947, which may be associated with poor seismic 87c monitoring capacity and record keeping. Since 1947, the number of shallow earthquakes per decade has continuously increased. The last decade generally reflects the region’s average seismic activity; therefore, its thermal radiation background information should be representative as well.

### 3.3. Earthquake Catalogue and Grouping

Table 1 lists 10 shallow earthquakes that occurred over the last decade, which can be divided into four chronological swarms. The first earthquake group has three earthquakes in 2008, including an Mw 6.1 single earthquake and an Mw 6.4 double shock. The second group has three earthquakes in 2010 and 2011, including an Mw 7.2 single earthquake and an Mw 6.7 main earthquake in 2010 and its Mw 6.2 aftershock in 2011. The third group has two earthquakes of Mw 7.7 and Mw 6.1 in the first half of 2013. The last earthquake group has two earthquakes in the second half of 2013, including an Mw 7.7 main earthquake and its Mw 6.8 aftershock.

## 4. Results

In this section, the wavelet filtering results and wavelet annual background anomaly analysis of Mw 7+ earthquake are first analyzed. Secondly, the short-term seismic thermal radiation anomalies of the four earthquake swarms are analyzed. Then, the long-term background anomaly characteristics of regional thermal radiation are examined. Finally, the effects of strong earthquakes on the regional thermal radiation environment are investigated.

### 4.1. Wavelet Filtering Results and Wavelet Annual Background Anomaly Analysis of Mw 7+ Earthquake

**Figure 5:**
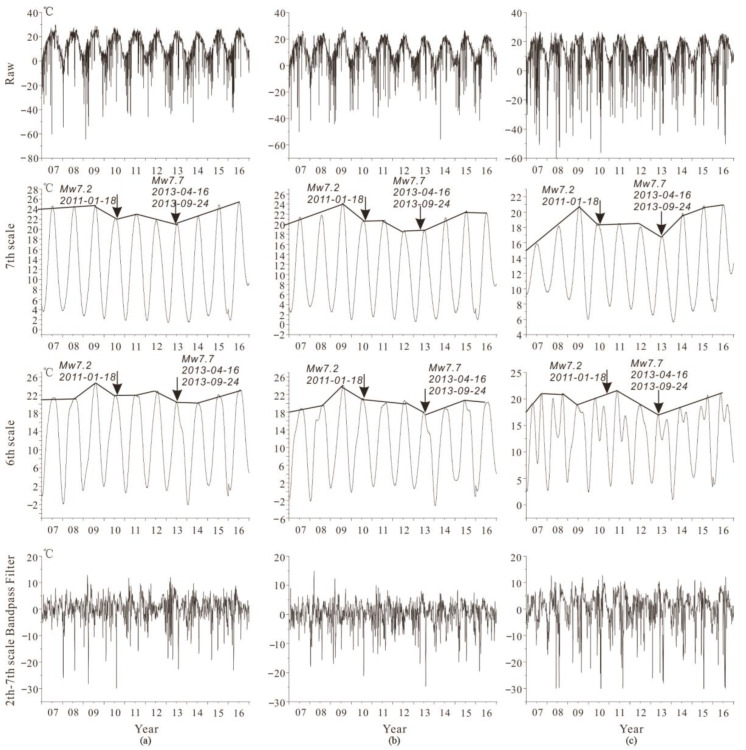
Different scale factor and band-pass filtering wavelet transform results for three Mw 7+ earthquakes. (**a**) Earthquake 1: 28.78° N, 63.95° E, Mw 7.2, 18 January 2011; (**b**) Earthquake 2: 28.03° N, 62.00° E, Mw 7.7, 16 April 2013; (**c**) Earthquake 3: 26.95° N, 65.50° E, Mw 7.7, 24 September 2013. Raw: raw data per day; 6th: sixth order; 7th: seventh order; 2nd–7th: second to seventh band-pass filter.

The positive–negative time domain waveform processed by the second- to seventh-order band-pass filtering can be separated from the low-frequency annual temperature field and the high-frequency temperature field caused by other short time factors (cloud, short time heating up or cooling down), as shown in Figure 5.

In Figure 5a, the raw data exhibit distinguishable annual variations caused by season alternation. Due to the short-term weather changes, the fluctuations of the data are relatively large, which can be handled by wavelet analysis. By applying the sixth and seventh order wavelet transform, it is possible to separate the approximate annual period (sixth scale) and annual period (seventh scale) change information, respectively. The seventh scale has a longer period, which is closer to one year. Figure 5b,c show that Mw 7+ can cause changes in the annual period (seventh scale) and approximate annual period (6th scale) background curves. Also, three earthquakes occurred in the background transition.

In conclusion, the second- to seventh-order band-pass Daubechies wavelet filtering is used to reduce noise of the low-frequency annual temperature field and the high-frequency temperature field caused by other short time factors. The sixth- and seventh-order scales indicate the annual background anomaly and correspond to the Mw 7+ earthquake time.

### 4.2. TFA of Earthquake Swarm Short-Term Thermal Radiation Anomalies

The TFRPS method was used to extract and identify the characteristic information of earthquake swarm thermal radiation anomalies. The analysis included the anomaly characteristic period, amplitude, duration, form, and the relationship among the anomaly range and earthquake epicenter and evolution characteristics. In the following section, the TFAs of the thermal radiation anomalies of the four earthquake swarms (G1–G4) are described in chronological order.

1. Short-term thermal radiation anomalies of G1

**Figure 6:**
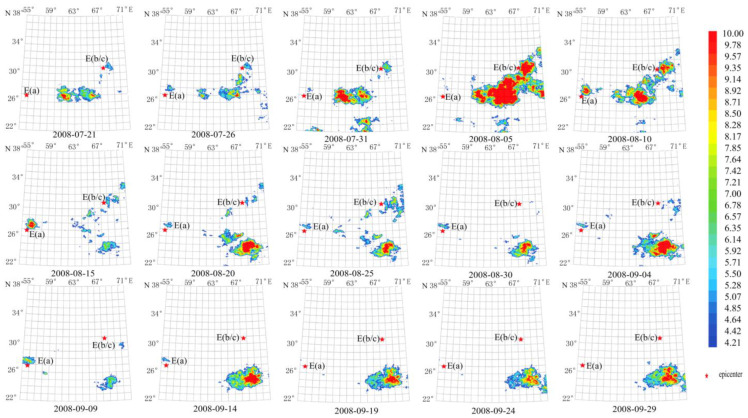
Temporal and spatial evolution of thermal radiation anomalies of earthquake group G1. Single earthquake: E(a) 26.74° N, 55.83° E, Mw 6.1, 10 September 2008. Double earthquake: E(b and c) 30.64° N, 67.35° E and 30.60° N, 67.40° E, Mw 6.4 and Mw 6.4 on 28 October 2008 and 29, respectively. Wavelet transform: second- to seventh-order band-pass filter using Daubechies wavelets. Window length of FT power spectrum: 64 days using Welch’s method. Characteristic period: 13 days.

As shown in Figure 6, the anomaly lasted about 70 days from 21 July 2008 to 29 September 2008. The maximum amplitude of the anomaly occurred on 5 August 2008, reaching more than 10 times the average value. The area of the anomaly was close to the double-shock epicenter, with the expansion direction nearly parallel to the line connection of these two epicenters (E(a), E(b)). The temporal and spatial evolution of the anomaly was clear and easy to extract and identify. The anomaly evolution presents a three-stage process of the abnormal occurrence–maximum–disappearance, in line with the earthquake propagation process.

2. Short-term thermal radiation anomalies of G2

**Figure 7:**
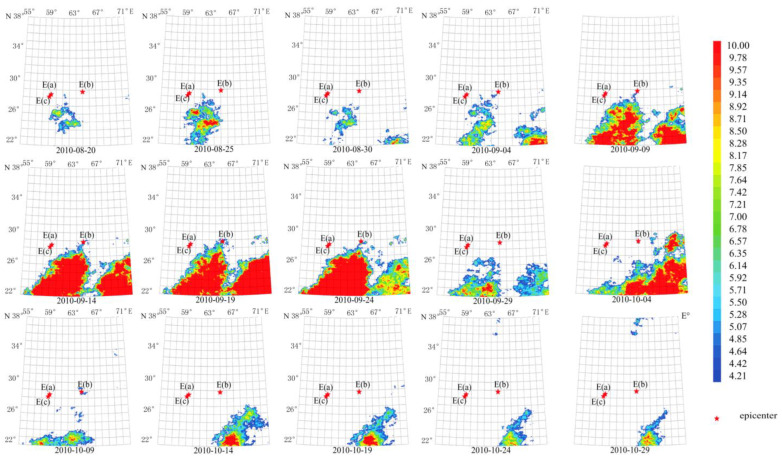
Temporal and spatial evolution of thermal radiation anomalies of earthquake group G2. Single earthquake: E(b) 28.78° N, 63.95° E, Mw 7.2, 18 January 2011. Main shock: E(a) 28.41° N, 59.18° E, Mw 6.7, 20 December 2010. Aftershock: E(c) 28.20° N, 59.02° E, Mw 6.2, 27 January 2011. Wavelet transform: second- to seventh-order scale band-pass filter using Daubechies wavelets. Window length of FT power spectrum: 64 days using Welch’s method. Characteristic period: 13 days.

The anomaly duration of these earthquakes was also about 70 days from 20 August 2010 to 29 September 2010, as shown in Figure 7. The maximum amplitude of the anomaly reached more than 10 times the average value on 14 September 2010. The anomaly center was close to the Mw 7.2 epicenter. The anomalous shape is consistent with the ShakeMap of Figure 2b, which is mainly located in the southern epicenter.

**Figure 8:**
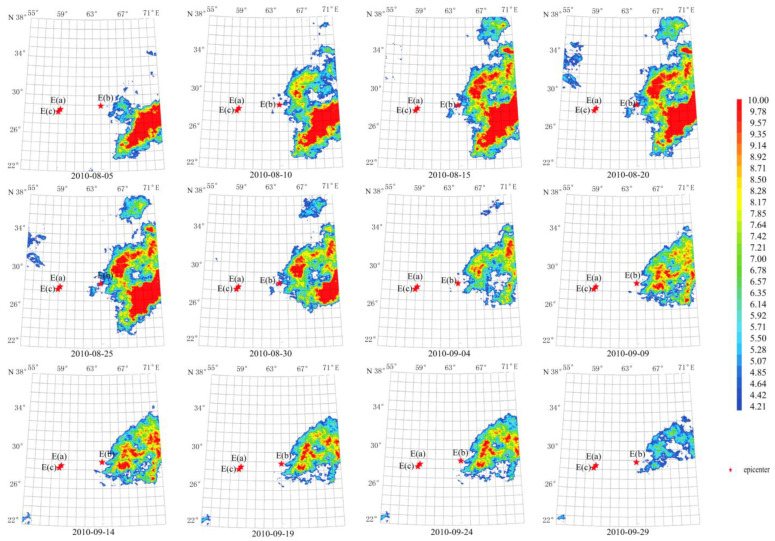
Temporal and spatial evolution of thermal radiation anomalies of earthquake group G2. Single earthquake: E(b) 28.78° N, 63.95° E, Mw 7.2, 18 January 2011. Main shock: E(a) 28.41° N, 59.18° E, Mw 6.7, 20 December 2010. Aftershock: E(c) 28.20° N, 59.02° E, Mw 6.2, 27 January 2011. Wavelet transform: second- to seventh-order scale band-pass filter using Daubechies wavelets. Window length of FT power spectrum: 64 days using Welch’s method. Characteristic period: 64 days.

Using a longer characteristic period of 64 days, temporal and spatial evolution of thermal radiation anomalies of earthquakes in group G2 demonstrates a pattern that lasted for about 60 days, as shown in Figure 8. The maximum amplitude of the anomaly occurred on 14 September 2010. The circular anomaly appears to the southeast of the epicenter and agrees with the ShakeMap in Figure 2b.

3. Short-term thermal radiation anomalies of G3

The anomaly, lasting about 60 days from 7 March 2013 to 1 May 2013, is elliptical with the major axis extending diagonally. The anomaly center lies on top of the E(a) epicenter, and its maximum amplitude occurred from 27 March 2013 to 16 April 2013, rising to about 10 times the average value. The area of the anomaly overlapped with the Mw 7.7 epicenter.

4. Short-term thermal radiation anomalies of G4

**Figure 9:**
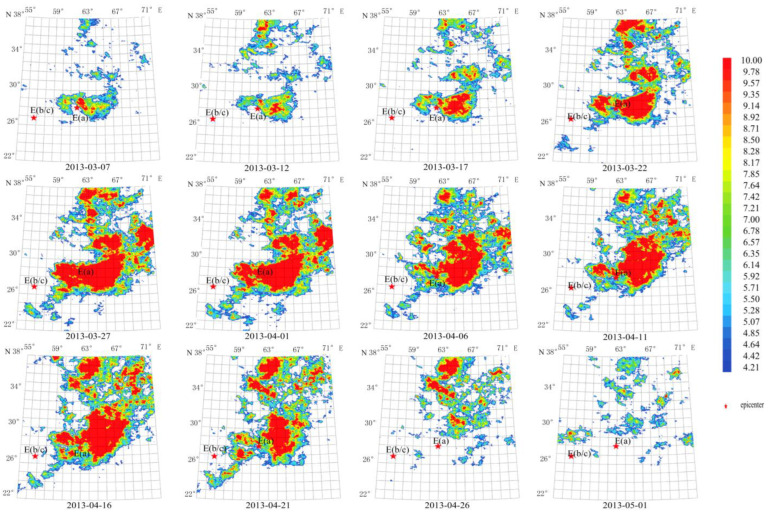
Temporal and spatial evolution of thermal radiation anomalies of earthquake group G3. Single earthquake: E(a) 28.03° N,62.00° E, Mw 7.7, 16 April 2013; E(b) 26.56° N, 57.77° E, Mw 6.1, 11 May 2013. Wavelet transform: second- and seventh-order band-pass filter using Daubechies wavelets. Window length of FT power spectrum: 64 days using Welch’s method. Characteristic period: 13 days.

The anomaly slightly extends from the epicenter to the southwest direction in an elliptical shape, which is consistent with Figure 2d. Its width along the south–north direction is shorter than the anomaly in Figure 9. The duration of this anomaly was about 55 days from 9 September 2013 to 3 October 2013. The maximum amplitude of the anomaly appeared during 14 August 2013 to 24 August 2013, reaching more than 10 times the average value.

**Figure 10:**
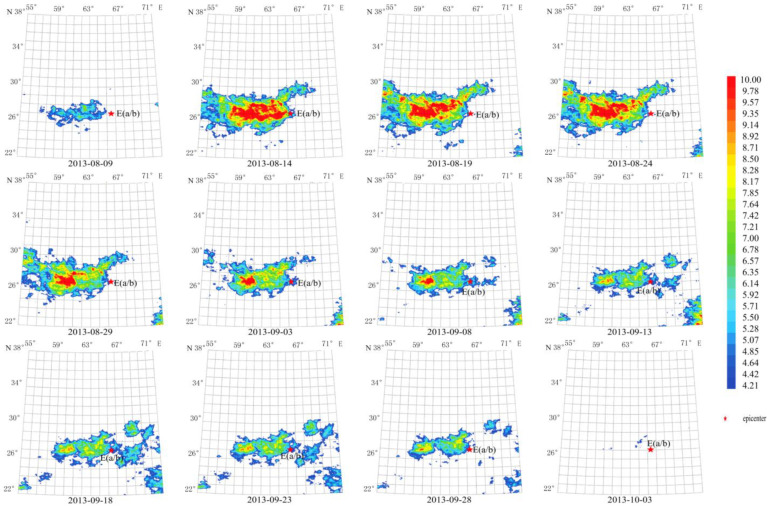
Temporal and spatial evolution of thermal radiation anomalies of earthquake group G4. Main shock: E(a) 26.95° N, 65.50° E, Mw 7.7, 24 September 2013. Aftershock: E(b) 27.18° N, 65.51° E, Mw 6.8, 28 September 2013. Wavelet transform: second- to seventh-order band-pass filter using Daubechies wavelets. Window length of FT power spectrum: 64 days using Welch’s method. Characteristic period: 64 days.

Compared to Figure 10, the anomaly in Figure 9, with a short characteristic period of 13 days, appeared 40 days pre-earthquake, reached the maximum when the earthquake occurred, and disappeared in 15 days after the earthquake. In addition, the anomaly area is much larger with the epicenter in the center, while in Figure 10, the anomaly appeared 45 days before the earthquake, and already started waning at the earthquake occurrence, and completely disappeared in the 10 days post-earthquake. The anomaly is more concentrated with the epicenter lying at its right edge.

As summarized in Table 2, short-term thermal radiation anomalies are irrelevant to the earthquake magnitude in terms of characteristic period, duration, and earthquake time and epicenter location. All anomalies lasted about 2 months and the earthquake occurred after they reached the maximum. Anomalies either overlap with or extend away from the epicenters. A positive relationship is identified between the anomaly morphology and the ShakeMap. That is, the higher the magnitude, the better they match each other.

In conclusion, the temporal and spatial evolution of the anomaly is clearly evident and can be easily extracted and identified. The anomaly evolution process presents a process of the appearance reaching the maximum disappearance, in line with the earthquake propagation process. The processes of the anomaly were not related to changes in weather but related to ShakeMap. Short-term seismic thermal radiation anomalies showed no obvious trends with earthquake magnitude, i.e., characteristic period, duration, earthquake time, and epicenter location are not positively related to earthquake magnitude. Strong earthquakes can affect regional thermal radiation background in that both the characteristic periods and the duration of impact became longer with increasing magnitude. By analyzing and summarizing the earthquake case, we found the following rules. The characteristic period of all earthquakes is 13 days if there is no earthquake of magnitude 7 within one year. If a magnitude 7 earthquake occurs within a year, the characteristic period of the earthquake in the region will increase from 13 days to 64 days. Therefore, we can use the short-term characteristic periods as an anomaly area indicator (AAI) to determine seismic anomalies.

### 4.3. Multiscale TFA of the Long-Term Thermal Radiation Anomalies of Mw 6+ Earthquake Swarms

In the following section, a multiscale TFA of the thermal radiation anomaly caused by large earthquakes is described in chronological order.

1. TF characteristics of long-term thermal radiation anomaly of Mw 6+ earthquakes in 2008

**Figure 11:**
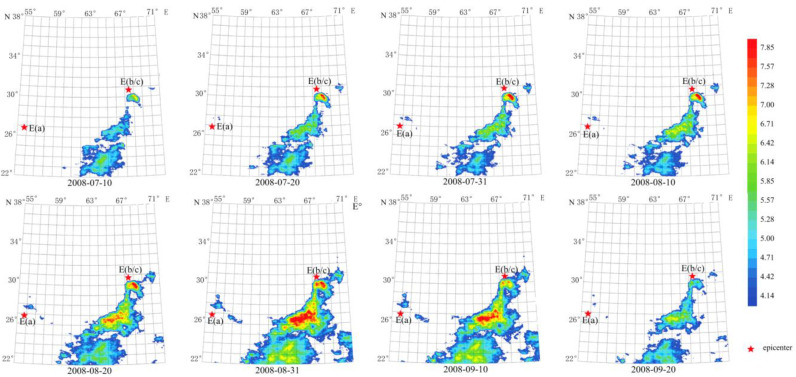
Temporal and spatial evolution of long-term thermal radiation anomaly of strong earthquakes. Single earthquake: E(a) 26.74° N, 55.83° E, Mw 6.1, 10 September 2008. Double earthquakes: E(b and c) 30.64° N, 67.35° E and 30.60° N, 67.40° E, Mw 6.4 and Mw 6.4, 28 October 2008 and 29, respectively. Wavelet transform: sixth order using Daubechies wavelets. Window length of FT power spectrum: 256 days using Welch’s method. Characteristic period: 128 days.

To extract the thermal radiation anomaly, the sixth-order transform using Daubechies wavelets and FT using Welch’s method with the window length of 256 days and the characteristic period of 128 days were conducted. As shown in Figure 11, the anomaly that arose 3 months pre-earthquake lasted about 70 days from 10 July 2008 to 20 September 2008. The maximum amplitude of the anomaly occurred on 31 August 2008, reaching more than eight times the average value. The anomaly was far from the epicenter of the first shock but close to the double shock epicenter. The temporal and spatial evolution of the anomaly was clear and matches the ShakeMap in Figure 2a. The anomaly evolution process presents a process of the abnormal occurrence–maximum–disappearance, in line with the earthquake propagation process. The processes of the anomaly were not related to changes in weather, indicating that it was caused by the earthquakes.

2. TF characteristics of long-term thermal radiation anomalies of Mw7+ earthquakes in 2010 and 2011.

**Figure 12:**
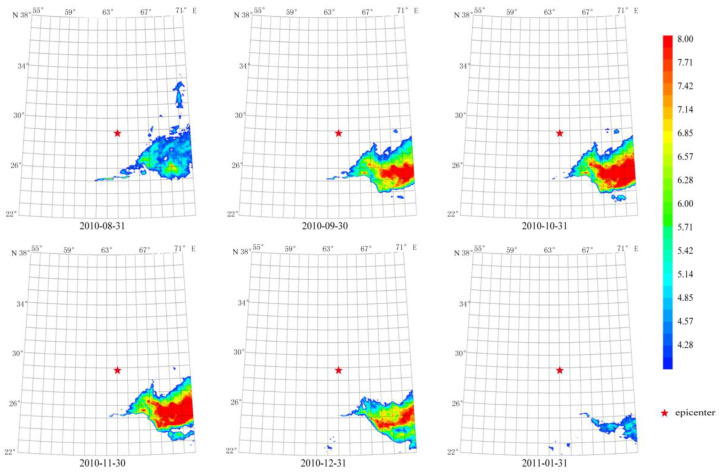
Temporal and spatial evolution of long-term thermal radiation anomaly of strong earthquakes. Single earthquake: E, 28.78° N, 63.95° E, Mw 7.2, 2011-01-18. Wavelet transform: sixth order using Daubechies wavelets. Window length of FT power spectrum using Welch’s method: 512 days. Characteristic period: 256 days.

As shown in Figure 12, this anomaly showed up about 5 months pre-earthquake, lasting from 31 August 2010 to 31 January 2011. It reached the maximum amplitude from 31 October 2010 to 30 November 2011, which is more than eight times the average value. The area of the anomaly was close to the Mw 7.2 epicenter, and its shape is similar to that of the ShakeMap in Figure 1b. Different from the short-term anomaly, this long-term one disappeared with the earthquake occurrence. Thus, it can indicate the earthquake in advance. In this case, both the sixth- and seventh-order wavelet transforms showed anomalies, and FT used the window length of 512 days and the characteristic period of 256 days.

**Figure 13:**
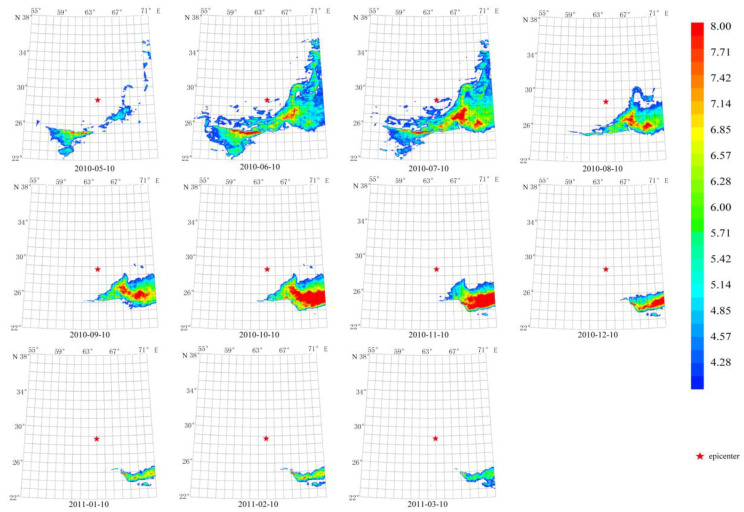
Temporal and spatial evolution of long-term thermal radiation anomaly of strong earthquakes. Single earthquake: E 28.78° N, 63.95° E, Mw 7.2, 18 January 2011. Wavelet transform: seventh order using Daubechies wavelets. Window length of FT power spectrum using Welch’s method: 512 days. Characteristic period: 256 days.

Figure 12 and Figure 13 are similar in anomaly morphology, amplitude, characteristic period, and evolution processes. Nonetheless, the anomaly from the seventh-order transform appeared 3 months earlier and lasted 3 months longer than that from the sixth order transform. In addition, the sixth order anomaly disappeared with the earthquake occurrence, while the seventh order one faded in 2 months post-earthquake.

3. TF characteristics of long-term thermal radiation anomalies of Mw 7+ earthquakes in 2013.

**Figure 14:**
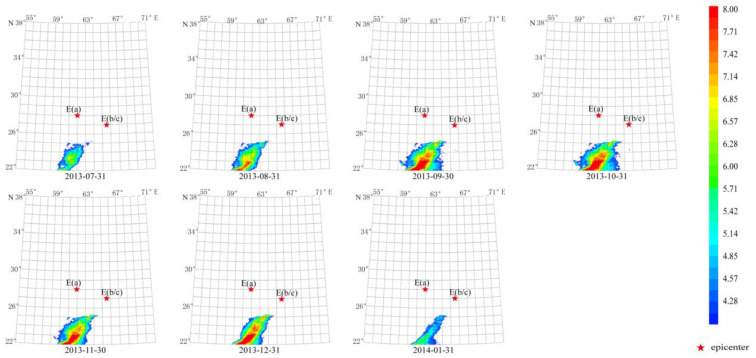
Temporal and spatial evolution of long-term thermal radiation anomaly of strong earthquakes. Single earthquake: E(a) 28.03° N, 62.00° E, Mw 7.7, 16 April 2013; E(b) 26.95° N, 65.50° E, Mw 7.7, 24 September 2013. Wavelet transform: sixth order using Daubechies wavelets. Window length of FT power spectrum using Welch’s method: 512 days. Characteristic period: 512 days.

**Figure 15:**
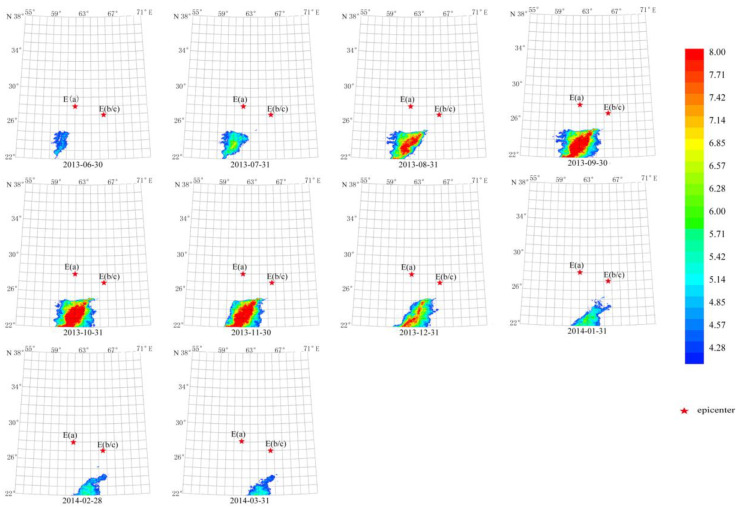
Temporal and spatial evolution of long-term thermal radiation anomalies of strong earthquakes. Single earthquake: E(a) 28.03° N, 62.00° E, Mw 7.7, 16 April 2013; E(b) 26.95° N, 65.50° E, Mw 7.7, 24 September 2013. Wavelet transform: seventh order using Daubechies wavelets. Window length of FT power spectrum using Welch’s method: 512 days. Characteristic period: 512 days.

Two Mw 7.7 earthquakes occurred separately in the first and second half of 2013 in this region. From Figure 9 and Figure 10, the characteristic period of the short-term anomaly elongated from 13 days to 64 days, indicating that the first Mw 7.7 earthquake changed the regional thermal radiation. The second earthquake, along with the first one, increased the characteristic period of the long-term anomaly to 512 days (Figure 14 and Figure 15).

The duration of the first anomaly was about 6 months from 31 July 2013 to 31 January 2014 (Figure 14). The maximum amplitude occurred from 30 September 2013 to 31 December 2013, which is eight times the average value. The second anomaly lasted about 9 months from 30 June 2013 to 31 March 2014. The maximum amplitude occurred from 30 September 2013 to 31 November 2013, which is also about eight times the average value. The shape of these two anomalies is similar to that of the ShakeMap provided by the USGS for the Mw 7.7 earthquakes (Figure 2d). These two anomalies showed similar characteristics, but their appearance and lasting time are obviously different. Specifically, the seventh-order anomaly appeared 1 month earlier and lasted 3 months longer than the sixth order one.

Long-term anomalies are positively correlated to the earth magnitude in that the higher the magnitude the longer the window length and characteristic period (Table 3). For earthquakes < Mw 7, the window length of relative power spectrum is 256 days; for Mw 7.0+ earthquakes, the window length is 512 days. Moreover, only the sixth-order transform shows an anomaly when the earthquake Mw is smaller than 7.0; while both the sixth- and seventh-order transforms show anomalies for Mw 7.0+ earthquakes. In addition, the anomalies of Mw 7.0+ earthquakes lasted 6 months or more and the anomaly duration from seventh-order transforms is longer than that from sixth-order transforms, but those of earthquakes < Mw 7.0 only lasted 2 months. Last, all anomalies reached their maximum before or at the earthquake occurrence.

In conclusion, long-term anomalies are positively correlated to magnitude, i.e., both the window length of RPS and characteristic period increased with increasing magnitude. In addition, for Mw 7.0− earthquakes, only the sixth-order wavelet transform showed anomalies, while both the sixth- and seventh-order wavelet transforms showed anomalies. Therefore, we can use the long-term characteristic periods and window lengths as a magnitude decision factor (MDF) to determine seismic magnitude.

## 5. Discussion

### 5.1. Earthquake Impact on Regional Thermal Radiation Background

1. Characteristic period of short-term anomalies

**Figure 16:**
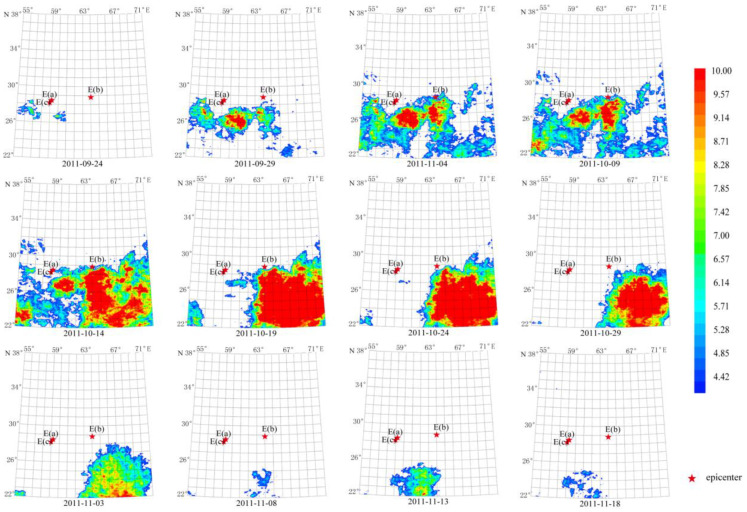
Spatial-temporal evolution of the thermal radiation background in 2011 caused by the Mw 7.2 earthquake. Single earthquake: E 28.78° N, 63.95° E, Mw 7.2, 18 January 2011. Wavelet transform: second- and seventh-order band-pass filter using Daubechies wavelets. Window length of FT power spectrum using Welch’s method: 64 days. Characteristic period: 64 days.

The periods of the thermal radiation anomalies were changed by Mw 7+ earthquakes. Earthquake group G2 consists of foreshock, mainshock, and aftershock. From August to October 2010, short-term thermal radiation anomalies with characteristic periods of 13 and 64 days appeared (Figure 7 and Figure 8). Later from September to November, another anomaly with the period of 64 appeared (Figure 16). Similarly, for G3 and G4, the characteristic period of the 16 April 2013 Mw 7.7 earthquake was 13 days but that of the 24 September 2013 Mw 7.7 earthquake increased to 64 days. However, Mw 6+ earthquakes from G1 do not show such an increasing trend in characteristic period. Therefore, the relative power spectrum of the 64-day characteristic period can be used to analyze the regional thermal radiation anomalies caused by strong earthquakes.

2. Duration of the impact and earthquake magnitude

**Figure 17:**
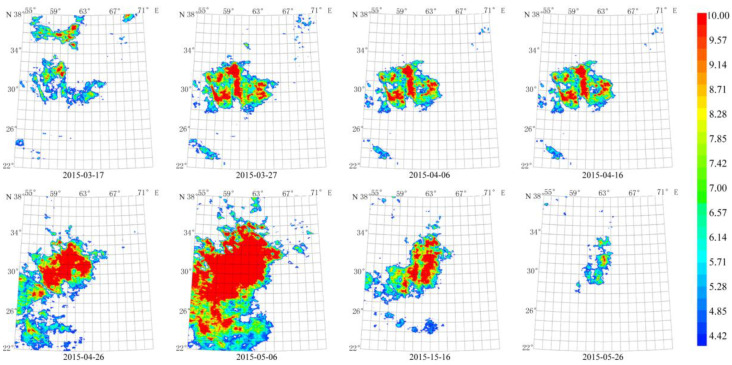
Spatial-temporal evolution of the thermal radiation background in 2015 caused by the Mw 7.7 earthquake. Earthquakes: E 28.03° N, 62.00° E, Mw 7.7, 16 April 2013; E 26.95° N, 65.50° E, Mw 7.7, 24 September 2013. Wavelet transform: second- and seventh-order band-pass filter using Daubechies wavelets. Window length of FT power spectrum: 64 days using Welch’s method. Characteristic period: 64 days.

**Figure 18:**
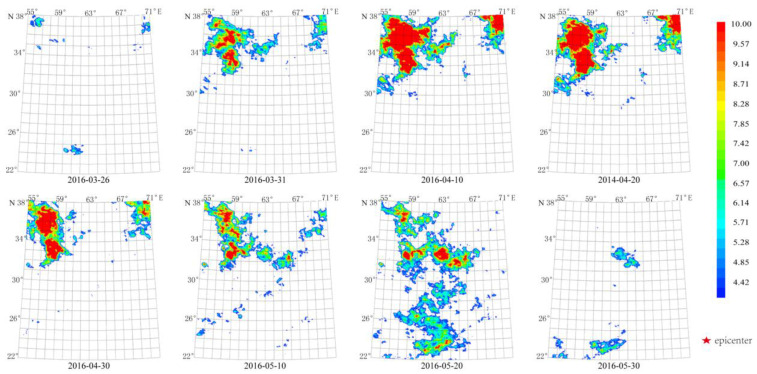
Spatial-temporal evolution of the thermal radiation background from 2016 caused by Mw 7.7 earthquakes. Earthquakes: E 28.03° N, 62.00° E, Mw 7.7, 16 April 2013; E 26.95° N, 65.50° E, Mw 7.7, 24 September 2013. Wavelet transform: second- and seventh-order band-pass filter using Daubechies wavelets. Window length of FT power spectrum: 64 days using Welch’s method. Characteristic period: 64 days.

Thermal radiation anomalies could be useful for studying strong earthquake processes and stress adjustment mechanisms. The 18 January 2011 Mw 7.2 earthquake affected the regional thermal radiation background for about 2 years (2010–2011), as shown in Figure 8 and Figure 16, while the 16 April 2013 Mw 7.7 and 24 September 2013 Mw 7.7 earthquakes had a 4-year effect (2013–2016), as shown in Figure 10, Figure 17 and Figure 18. The duration of the impact increases with the magnitude of the earthquake, which may be further related to the regional stress adjustments and recovery post-earthquakes. Thus, longer duration of the impact could probably indicate slower regional stress adjustment and recovery.

By carrying out the TFA for the past 10 years’ data with the characteristic period of 64 days, we found the thermal radiation anomaly caused by the Mw 7.2 earthquake impact lasted 2 years from 2010 to 2011. The duration of the anomaly was about 55 days from 24 September 2011 to 18 November 2011. The maximum amplitude of the anomaly occurred from 14 October 2011 to 29 October 2011, reaching more than 10 times the average value. The anomalous area was closer to the Mw 7.2 epicenter. The long-term and concentrated thermal radiation anomalies were similar to those of the Mw 7.2 earthquake group in terms of characteristic period, amplitude, form, and evolution.

From the data from 2013 to 2016, we found the thermal radiation anomaly caused by the double Mw 7.7 earthquakes. Their impact lasted for 4 years from 2013 to 2016. The anomaly continued for about 70 days from 17 March 2015 to 26 May 2015, which is consistent with the anomaly of the 16 April 2011 Mw 7.7 earthquake. The maximum amplitude of the anomaly that is more than 10 times the average value occurred from 26 April 2015 to 16 May 2015. The anomaly was close to the double Mw 7.7 epicenter. In addition, the long-term and concentrated thermal radiation anomalies are similar to those of the 16 April 2013 Mw 7.7 earthquake in terms of amplitude, morphology, and evolution (Figure 17).

The anomaly from 26 March 2016 to 30 May 2016 with the maximum amplitude occurring from 10 April 2016 to 30 April 2016 was also closer to the double Mw 7.7 epicenter (Figure 18) and similar to that of the 16 April 2013 Mw 7.7 earthquake. The thermal radiation anomalies of the 24 September 2013 Mw 7.7 earthquake are different from that of the 2015–2016 in morphology. Specifically, the thermal radiation anomalies from the 24 September 2013 Mw 7.7 earthquake were concentrated at the epicenter and nearby areas, and the area of the anomaly was smaller (Figure 10) than that of the anomaly from 2015 (Figure 17), and less dispersed than that of the anomaly from 2016 (Figure 18). The absence of thermal radiation in 2014 might be a consequence of the Mw 7.7 earthquake in September 2013.

3. Superposition of short-term anomalies induced by multiple earthquakes

If more than one Mw 6.0+ earthquake occurred over a 1-year time scale, the characteristics of the seismic thermal radiation anomalies became complex and were difficult to extract and identify, which is due to the superposition of the regional thermal radiation caused by the mutual coupling between strong earthquakes. The thermal radiation anomalies of the 2010–2011 earthquake swarms were complex, and the characteristic periods of 13 and 64 days appeared simultaneously, possibly because the earlier Mw 7.2 earthquake changed the latter regional thermal radiation. In addition, the characteristic period of the 16 April 2013 Mw 7.7 earthquake was 13 days, whereas that of the 24 September 2013 Mw 7.7 earthquake was 64 days, which may also have been affected by the earlier 16 April 2013 Mw 7.7 earthquake. This is also supported by the fact that there were no thermal radiation anomalies in March–May 2014.

### 5.2. Relationship between Thermal Anomalies and Earthquake Magnitude

From Table 2, short-term seismic thermal radiation anomalies showed no obvious change with earthquake magnitude, indicating that this method is suitable for extracting anomaly characteristics. In addition, characteristic period, duration, earthquake time, and epicenter location are not positively related to earthquake magnitude. All anomalies endured about 2 months, with the maximum amplitude appearing before the earthquake. The positive correlation identified is that the higher the magnitude, the better correspondence between the anomaly morphology and the ShakeMap.

Table 3 suggests strong relationships between long-term anomaly characteristics and earthquake magnitude. Since generally the longer the energy accumulates, the higher the earthquake magnitude, a longer characteristic period indicates longer energy accumulation time. This finding could serve as a criterion for determining the earthquake magnitude. In terms of wavelet scale, if only the sixth-order transform shows anomaly, the earthquake magnitude can be determined to be Mw 7.0−; if both the sixth- and seventh-order transforms show anomalies, the magnitude can be determined to be Mw 7.0+. Moreover, the longer the characteristic period, the higher the earthquake magnitude. Specifically, a period of 256 days corresponds to Mw 7.5−, and a period of 512 days indicates Mw 7.5+ earthquakes.

## 6. Conclusions

In this paper, we analyzed the characteristics of seismic thermal radiation anomalies in the same area. The results of this study have found the regular pattern and mechanism of anomaly seismic thermal radiation in the same region, and also improved the accuracy of the analytical processing model. The MS T-FRPS method was used to identify and determine the anomalous information of series earthquake groups with consistency law, which proves the stability and reliability of the method. By analyzing and summarizing the earthquake case, we propose the anomaly area indicator (AAI) to determine seismic anomalies using short-term characteristic periods and the magnitude decision factor (MDF) to determine seismic magnitude using the long-term characteristic periods and window lengths. In addition, we have explored the following: earthquake impact on regional thermal radiation background and relationship between thermal anomalies and earthquake magnitude and the like. Future research directions using the MS T-FRPS method to characterize regional thermal radiation anomalies induced by strong earthquakes could be (1) carrying out more case studies with accurate parameter quantification; (2) studying the relationship between thermal radiation anomalies and the earthquake magnitude; (3) employing methodologies where the correlation and causality between spatial points is examined in other phenomena; (4) utilizing machine learning algorithms to analyze earthquake precursors, crustal movements, and other data to enhance the accuracy of earthquake predictions. Meaningful findings could help improve the accuracy of earthquake magnitude determination.

## Figures and Tables

**Table 1:** Earthquake catalogue and grouping.

Date	Latitude (°)	Longitude (°)	Depth (km)	Magnitude and Type	Place	Group
10 September 2008	26.74	55.83	12	6.1 mwc	Southern Iran	G1
28 October 2008	30.64	67.35	15	6.4 mwc	Pakistan	
29 October 2008	30.60	67.46	14	6.4 mwc	Pakistan	
20 December 2010	28.41	59.18	12	6.7 mwc	Southeastern Iran	G2
18 January 2011	28.78	63.95	68	7.2 mww	Southwestern Pakistan	
27 January 2011	28.20	59.06	10	6.2 mww	Southeastern Iran	
16 April 2013	28.03	62.00	80	7.7 mww	83 km E of Khash, Iran	G3
11 May 2013	26.56	57.77	15	6.1 mww	94 km SE of Minab, Iran	
24 September 2013	26.95	65.50	15	7.7 mww	61 km NNE of Awaran, Pakistan	G4
28 September 2013	27.18	65.51	12	6.8 mww	85 km NNE of Awaran, Pakistan	

**Table 2:** A summary of short-term thermal radiation anomalies of earthquakes.

Magnitude	Characteristic Period	Relationship of Abnormal Morphology and ShakeMap	Epicenter Location	Duration	Earthquake Time
Double Mw 6.4 28&29 October 2008	13 Days	Inconsistency	Abnormal edge	about 2 months	2 months after the maximum anomaly
Mw 6.7 20 December 2010	13 Days	Consistency	Abnormal edge	about 2 months	1 month after the maximum anomaly
Mw 7.2 18 January 2011	64 Days	Consistency	Abnormal edge	about 2 months	2 months after the maximum anomaly
Mw 7.7 16 April 2013	13 Days	Very consistent	Abnormal center	about 2 months	20 days after the maximum anomaly
Mw 7.7 24 September 2013	64 Days	Very consistent	Abnormal edge	about 2 months	1 month after the maximum anomaly

**Table 3:** A summary of the relationship between seismic anomaly and magnitude.

Magnitude	Wavelet Scale	Window Length of RPS	Characteristic Period	Duration	Earthquake Time
Mw 6.4	Only 6th	256 Days	128 Days	About 2 months	2 months after the maximum anomaly
Mw 6.7 Mw 7.2	6th and 7th	512 Days	256 Days	About 5 months (6th)	2 months after the maximum anomaly
				About 10 months (7th)	3 months after the maximum anomaly
Two Mw 7.7	6th and 7th	512 Days	512 Days	About 6 months (6th)	When the maximum anomaly occurred
				About 9 months (7th)	When the maximum anomaly occurred

## Data Availability

Data sharing is not applicable to this article as no datasets were generated or analyzed during the current study.
